# Study of the influence of exogenous fibrolytic enzyme additive on chemical composition, fermentation characteristics, and nutritional value of brewer’s spent grain

**DOI:** 10.1002/fsn3.2743

**Published:** 2022-01-23

**Authors:** Khalil Abid, Jihene Jabri, Hela Yaich, Atef Malek, Jamel Rekhis, Mohamed Kamoun

**Affiliations:** ^1^ Animal Nutrition Laboratory National School of Veterinary Medicine Sidi Thabet University of Manouba Sidi Thabet Tunisia

**Keywords:** Brewer's spent grain, chemical composition, exogenous fibrolytic enzyme, fermentation characteristics, nutritional value

## Abstract

This study explores the influence of different doses of two exogenous fibrolytic enzyme (EFE) additives (liquid (EFE_1_: 1, 2, and 4 μΙ/g DM (dry matter)) and powder (EFE_2_: 1, 2, and 4 mg/g DM)) on the chemical composition, fermentation characteristics, and nutritional value of brewer's spent grain (BSG). The results indicate that EFE_1_ at low doses does not affect the chemical composition, fermentation characteristics, and the nutritional value of BSG. The medium dose EFE_1_ decreases the fiber compound but increases the nonfiber carbohydrates (NFC) and soluble dry matter. Also, this dose modified the fermentation of BSG by increasing the amount of gas and its fermentation rate and decreasing the time between the inoculation and start of fermentation. Therefore, it increases the digestibility, metabolizable energy, net energy‐lactation (NE_L_), total volatile fatty acids, and the microbial crude protein production of BSG. The high dose of EFE_1_ decreases the fiber compound and increases the nonfiber carbohydrates and soluble dry matter; however, it also decreases the potential of gas production and does not affect the nutritional value of BSG. For EFE_2_, all the doses do not modify the chemical composition, fermentation characteristics, and the nutritional value of BSG. These results suggest that the effectiveness of EFE varied, depending on the type of EFE and dose. Increase in the nutritional value of BSG by EFE_1_ at the medium dose can encourage breeders to use these wastes as feed at low cost in cow nutrition.

## 1 INTRODUCTION

The use of agro‐industrial wastes as ruminant feed is an eco‐friendly solution to reduce pollution and protect the environment (Kholif et al., 2017; Wanapat et al., 2018). Brewer's spent grain (BSG) constitutes approximately 85% of the brewing waste weight produced by the brewing industry, 31% of the malt weight, and 20 kg per 100 L of beer. The annual worldwide production of BSG was estimated to be about 39 million tons (Mussatto et al., 2006, 2014; Lynch et al., 2016). This waste spoils rapidly and can create a great amount of ecological problems (Lazarevich & Lesnov, 2010). This solid by‐product is a mixture of insoluble malted seed and seed coat—pericarp husk—of barley grain (Lynch et al., 2016). Fibers and proteins compounds are the principal compound of this agro‐industrial wastes (Lynch et al., 2016; Mussatto et al., 2006). Due to their relatively low price, approximately 39 USD∙per ton, BSG is used for as nutrition for ruminants ((Lynch et al., 2016). It has been found to increases milk yield, without affecting animal fertility (Mussatto et al., 2006) and also provides a wide variety of amino acids that are essential in the diet of cows (Lynch et al., 2016). Although the capacity of ruminants to degrade fibrous feed by the rumen microbes is inherent, this digestion is partial in most cases as only 10%–35% of gross energy is used as net energy (Varga & Kolver, 1997). To ameliorate the nutritional value of fiber feed, several approaches had been used, such as the chemical additive and biological additive. The biological treatments are more effective than the chemical treatments due to their greater substrate specificity and their lower pollution effects (Misra et al.,  2006). Several studies show that the use of EFE enhanced digestibility of fibrous feed (Abid et al., 2019, 2020), growth performance, and milk production and economic returns in the dairy industry (Abid et al., 2020; Lunsin et al., 2021; Mohamed et al., 2013).

The aim of this work is to explore the influence of different doses of two EFEs on the chemical composition, fermentation characteristics, and nutritional value of BSG.

## 2 MATERIALS AND METHOD

### 2.1 Sampling and treatment

Fresh BSG was collected from the beer industry located in Tunis (North of Tunisia). BSG was treated with two exogenous fibrolytic enzymes (EFEs) (EFE_1_: 1, 2, and 4 μΙ/g DM (dry matter) and EFE_2_: 1, 2, and 4 mg/g DM) at 26°C and for 12 h before the in vitro fermentation. EFE_1_ is a liquid mixture (50:50) of Cellulase Plus and Xylanase Plus (Dyadic International Inc. Jupiter, Florida) from *T*. *longibrachiatum*. The EFE_2_ is a powder preparation (MAXFIBER−I^®^, Shaumann GmbH, Wahlstedt, Germany) from *A*. *niger*, *A*. *tubingensis*, *A*. *orzyae*, *A*. *sojae*, and *N*. *intermedia*. These enzyme activities were measured at a pH of 6.6 and a temperature of 39°C, which imitate a cow rumen environment. The endoglucanase and exoglycanase were analyzed using cellulose and carboxymethylcellulose sodium salt as the substrate, conferring to the methods defined by Wood and Bhat (1988). Xylanase activity was tested using oat spelt xylan as the substrate according to the methods of Bailey et al., 1992. The enzyme activity of EFE1 and EFE2 is noted in Table 1.

**TABLE 1:** Enzymatic activity of the exogenous fibrolytic enzyme (EFE) preparation used in experiments (in international unit)

Enzyme preparation	Endoglucanase	Xylanase	Exoglucanase
EFE_1_	1554	2573	160
EFE_2_	750	1180	740

### 2.2 Chemical analyses

Samples of BSG untreated and treated with the appropriate EFE and in various doses were oven‐dried to constant weight aimed to determine dry matter (DM) content and then were ground to 1 mm (Cyclotec 1093 Sample Mill; Tecator, Höganäs, Sweden). Before that, the sample was examined to analyze crude protein (CP), ether extracts (EE), and ash conferring to the procedures described by the Association of Official chemists Analytical Chemists (1995). Neutral detergent fiber (NDF), acid detergent fiber (ADF), and acid detergent lignin (ADL) were tested by using a fiber analyzer (ANKOM 220, ANKOM Technology, Macedon, NY) according to the technique defined by Van Soest et al. (1991). The soluble dry matter (DMS) was estimated following the protocol of Elwakeel et al. (2007). Nonfiber carbohydrates (NFC) are estimated with the formula of the National Research Council (NRC) (2001) (Equation 1):

(1) $$\text{NFC}\;\left(\%\right)=100‐\left(\text{EE}\;(\%)+\text{CP}\;(\%)+\text{NDF}\;(\%)+\text{Ash}\;(\%)\right)$$

### 2.3 In vitro ruminal fermentation

Rumen fluid for fermentation studies was collected from two Holstein cows (650 ± 20 kg) fed twice daily with a diet of 8 kg of grass hay and 2 kg of concentrate. The rumen content was filtered through four layers of cheesecloth, mixed with a buffer solution (1:2 v/v), and held under a continuous flow of CO_2_ (Menke & Steingass, 1988). Samples of 200 mg DM of BSG (untreated and treated with the appropriate EFE and doses) and 30 ml of the incubation inoculum were incubated in 120‐ml volume serum bottles. Blank samples (negative controls) were used to remove for gas production from the fermentation of residues. The bottles were instantly closed with rubber stoppers and placed in a shaking water bath at 39°C for 96 hr. This research was realized at three runs and three repeats per run. Gas pressure was recorded at 2, 4, 6, 8, 12, 24, 48, 72, and 96 hr by a pressure transducer related to a visual display transducer.

Kinetic gas production was estimated according to the model of France et al. (2000), Equation 2, by using the nonlinear model of the SAS Institute Inc. (2011):

(2) $${\text{GP}}_{\left(t\right)}=B\;\left(1‐e^{‐C(t‐Lag)}\right),$$

where GP is the cumulative gas produced at the time t (ml/g DM), t is the incubation time (h), B is the potential of gas production (ml/g DM), C is the rate of gas production (ml/h), and Lag is time between inoculation and commencement of gas production (h).

The organic matter degradability (OMD), metabolizable energy (ME) values, and net energy‐lactation (NE_L_) were calculated with Equations 3, 4, and 5, obtained from the work of Menke and Steingass (1988). The total volatile fatty acids (VFA) were estimated with Equation 6, which was referred from Getachew et al. (1998):

(3) OMD=14.88+0.889×GP+0.45×CP+0.0651×Ash,

(4) ME=2.2+0.136×GP+0.0057×CP,

(5) NEl=0.101×GP+0.051×CP+0.112×EE,

(6) VFA=‐0.00425+0.0222×GP,

where OMD is organic matter degradability in %, ME is the metabolizable energy value in MJ/kg dry matter, NE_L_ is net energy‐lactation (NE_L_) in MJ/kg dry matter, VFA are the ruminal total volatile fatty acids in mmol/200 mg dry matter, GP is the net gas production (ml) from 200 mg after 24 hr of incubation, and CP is the crude protein in % dry matter, ash in % dry matter, and EE is ether extracts in % dry matter.

At the end of fermentation, bottles were put in ice for 5 min and the fermentation was stopped. The dry matter degradability in % (DMD) was measured according to the protocol of Elghandour et al. (2018). The partitioning factor of incubation at 24 hr (PF 24) (to estimate the efficiency of fermentation)) and the microbial crude protein (MCP) were determined with the equation of Blümmel et al. (1997):

(7) $$\text{PF}24=\frac{\text{aDMD}}{\text{GP}}$$

where PF 24 is the partitioning factor of incubation at 24 hr, GP is the net gas production in milliliters (ml) from 1 g of DM at 24 hr of fermentation, and aDMD is the amount of dry matter digestibility in grams at the end of incubation.

(8) MCP=aDMD‐2.2×GP,

where MCP is the microbial crude protein in mg/g dry matter, GP is the net gas production (ml) from 200 mg of DM of substrate at 24 hr of fermentation, aDMD is the amount of dry matter digestibility in g at the end of incubation, and the 2.2 mg/ml is a stoichiometric factor (Blümmel et al., 1997).

### 2.4 Statistical analysis

Data were evaluated via the general linear method (GLM) of the SAS Institute Inc. (2011) (Equation 9):

(9) $$Y_{\text{ijk}}=\mu +D_{i}+{\text{EFE}}_{j}+{\left(D\times \text{EFE}\right)}_{\text{ij}}+E_{\text{ijk}},$$

where *μ* is the overall mean, *D*_i_ is the effect of the dose (*i* = 0, 1, 2, and 4), EFE_j_ is the effect of the EFE (*j* = 1, 2), (*D* × EFE)_ij_ is the interaction between the dose and the EFE, and E_jik_ is the error term.

The orthogonal contrasts were performed to study the linear and quadratic properties of doses for each EFE. The difference between the mean was evaluated by using Duncan's tests with *p* value<0.05 (Duncan, 1955).

## 3 RESULTS

The influence of EFF on the chemical composition of BSG is presented in Table 2. The effectiveness of EFE varied with the type of EFE, dose of EFE, and their interaction. The EFE_1_ linearly decreases NDF and NDF contents and increases NFC and SDM. For EFE_2_, all doses do not affect the chemical composition of BSG.

**TABLE 2:** Influence of exogenous fibrolytic enzymes (EFEs) on the chemical composition of Brewer's spent grain (BSG) after preincubation (% dry matter)

Item	EFE1						
	0	1	2	4	*SEM*	Linear	Quadratic
DM	22.2	20.2	20.1	20.1	0.2	0.94	NS0.95
CP	28.9	28.9	28.9	29.0	0.3	0.98	0.99
NDF	48.2^a^	48.0^a^	42.3^b^	44.2^b^	2.1	0.04	0.24
ADF	20.9^a^	21.0^a^	18.1^b^	17.9^b^	1.8	0.03	0.33
ADL	6.7	6.5	6.4	6.5	0.7	0.89	0.91
EE	9.9	10.0	9.9	10.1	0.5	0.90	0.88
Ash	4.1	4.2	3.9	4.0	0.7	0.89	0.88
NFC	8.9^b^	8.9^b^	15^a^	12.7^a^	1.9	0.04	0.09
DMS	5.2^b^	5.3^b^	9.4^a^	9.2^a^	0.9	0.02	0.16

	EFE2						
	0	1	2	4	*SEM*	Linear	Quadratic
DM	22.2	22.2	22.1	22.3	0.3	0.99	0.92
CP	28.9	29.1	28.9	29.0	0.4	0.93	0.99
NDF	48.2	48.0	48.1	47.9	2.0	0.97	0.91
ADF	20.9	20.8	20.8	20.7	1.6	0.89	0.91
ADL	6.7	6.6	6.8	6.8	0.7	0.89	0.87NS
EE	9.9	9.9	10.0	10.2	0.7	0.91	0.88
Ash	4.1	4.2	4.0	4.2	0.7	0.87	0.81
NFC	8.9	8.8	9	8.7	2.0	0.94	0.93
DMS	5.2	5.7	5.6	5.7	1.0	0.70	0.67

*p*‐value			
	Dose	EFE	Dose ×EFE
DM	0.89	0.88	0.88
CP	0.9	0.91NS	0.9NS
NDF	0.02	0.03	0.03
ADF	0.03	0.03	0.03
ADL	0.90	0.91	0.91
EE	0.91	0.91	0.91
Ash	0.89	0.85	N0.87
NFC	0.04	0.03	0.04
DMS	0.04	0.02	0.04

Abbreviations: ADF, acid detergent fiber; ADL, acid detergent lignin; CP, crude protein; DM, dry matter; DMS, soluble dry matter; EE, ether extracts; NDF, neutral detergent fiber; NFC, nonfiber carbohydrates; NS, not specified; *SEM*, standard error of the mean.

^a,b,c^ Means in the same row with different superscripts differed (p < .05).

The influence of EFE on fermentation characteristics and nutritional value of BSG varied with the type of EFE, dose of EFE, and their interactions (Table 3). Only the medium dose of EFE1 modified the kinetics (improved B (potential of gas production (ml/g DM) and C (rate of gas production (ml/h), decreased lag) and improved ME, NE_L_, VFA and OMD, of BSG. However, exercise dose decreases nutritional value and fermentation characteristics of BSG. For EFE_2_, all doses do not affect fermentation characteristics and the nutritional value of BSG.

**TABLE 3:** Effect of exogenous fibrolytic enzymes' (EFEs') treatment on parameters of kinetics of fermentation and nutritional value of brewer's spent grain (BSG)

Item	EFE1						
	0	1	2	4	*SEM*	Linear	Quadratic
B	176.5^ b^	172.4^ b^	194.9^ a^	166.8^c^	4.7	0.44	0.009
C	0.045^b^	0.044^b^	0.071^a^	0.049^b^	0.018	0.31	0.02
Lag	1.01^b^	0.99 ^b^	0.12^a^	1.01 ^b^	0.07	0.62	0.007
OMD	56.4^b^	57.5^b^	62.4^a^	56.6^b^	2.10	0.60	0.01
DMD	54.2	55.1	57.2	56.3	4.7	0.41	0.08
ME	6.69^b^	6.85^b^	7.60^a^	6.72^b^	0.30	0.60	0.01
EN_L_	5.79^b^	5.93^b^	6.47^a^	5.84^b^	0.27	0.58	0.009
VFA	0.701^b^	0.728^b^	0.850^a^	0.706^b^	0.100	0.06	0.01
PF24	3.41	3.34	3.08	3.52	0.41	0.23	0.09
MCP	472.1^b^	478.4^b^	507.3^a^	492.6^a^	16.7	0.04	0.008

	EFE2						
	0	1	2	4	*SEM*	Linear	Quadratic
B	176.5	176.6	179.4	179.9	4.0	0.32	0.76
C	0.045	0.044	0.042	0.050	0.011	0.76	0.41
Lag	1.01	1.02	1.00	1.10	0.08	0.30	0.77
OMD	56.4	57.1	57.2	57.0	2.08	0.77	0.80
DMD	54.2	53.9	54.4	55.6	4.2	0.84	0.65
ME	6.69	6.79	6.81	6.77	0.29	0.77	0.80
EN_L_	5.79	5.88	5.90	5.89	0.23	0.77	0.80
VFA	0.701	0.717	0.722	0.715	0.009	0.77	0.80
PF24	3.41	3.33	3.33	3.37	0.99	0.74	0.71
MCP	472.04	469.5	472.06	474.72	16.5	0.80	0.80

*p*‐value			
	Dose	EFE	Dose ×EFE
B	0.04	0.03	0.04
C	0.04	0.02	*0.03
Lag	0.03	0.003	0.01
OMD	0.04	0.04	0.04
DMD	NS	NS	NS
ME	0.04	0.04	0.04
EN_L_	0.04	0.04	0.04
VFA	0.04	0.04	0.04
PF24	0.67	0.54	0.58
MCP	0.02	0.03	0.03

Note

NS: *p* >.05; *: *p* <.05, **: *p* <.01

Abbreviations: B, potential of gas production (ml/g DM); C, rate of gas production (ml/h); DMD, dry matter degradability (%); Lag, time between inoculation and commencement of gas production (h); MCP, microbial crude protein (mg/g DM); ME, metabolizable energy value (MJ/kg DM); NE_L_, net energy‐lactation (MJ/kg DM); NS, not specified; OMD, organic matter degradability (%); PF24, partitioning factor of incubation at 24 hr (mg substrate truly degraded/mL gas);*SEM*, standard error of the mean; VFA, ruminal total volatile fatty acids (mmol/200 mg DM).

^a,b,c^ Means in the same row with different superscripts differed (p < .05).

## 4 DISCUSSIONS

The chemical compositions of BSG were relatively similar for the chemical composition of BSG in the review realized by Lynch et al. (2016). This waste was characterized by high CP (>28%), which was corrected protein‐deficiency ruminants’ ratio, high NDF compound (>40%), and fat content (>9%). The high fat content may decrease the digestibility of carbohydrates and limit the connection of cellulosic bacteria to feed particles (Clinquart et al., 1995).

The effectiveness of the EFE depends on the type of EFE preparation. Indeed, EFE_2_ did not have any significant impact on the chemical composition, parameters of kinetics of fermentation, and nutritional value of BSG. The lack of effect of EFE2 can also be explained by the fact that the enzyme activity of this preparation was incompatible with the substrate used, or/and the doses used are not efficient, or/and the form of this enzyme (powder) was not very active. According to Beauchemin et al. (2004) and Beauchemin and Holtshausen (2010) water services, the diffusion of enzymes is vital for the hydrolysis of fibrous polymers.

The effectiveness of the EFE depends on the dose. The low dose of EFE_1_ did not affect chemical composition, parameters of kinetics of fermentation, and nutritional value of BSG.

The medium dose decreases NDF and NDF contents and increases nonfiber carbohydrates and SDM. This effect is due to the breakdown of the link between hemicellulose, cellulose, and lignin (Han et al., 2007; Morais et al., 2017). These consequences were analogous to those described by Díaz et al. (2015) on fibrous feeds such as *Dichanthium aristatum* and by Abid et al. (2019) on by‐products such as almond hull and pomegranate hull. Consequently, it increased the potential of gas production by 10% due to more available nonfiber carbohydrates for rumen microorganisms (Makkar, 2010; McDonald et al., 2011). A similar trend was found by Abid et al. (2019) for the almond hull. In addition, this dose increases the rate of fermentation from 0.045 to 0.071 ml/h. These improvements in the rate of fermentation may reduce the period of stay of feed in the rumen and induce greater dry matter intakes. Also, it decreases the time between inoculation and the beginning of fermentation from 1.01 to 0.12 h. A similar result was found by Yang et al. (1999) and Wang et al. (2001). On the other hand, this additive at medium dose increased the degradability of organic matter by 11%. A similar result was found by Abid et al. (2019) on almond hull. Moreover, this treatment at medium dose increases the ME from 6.5 to 7.2 MJ/kg DM. Therefore, this treatment makes the ME of this by‐product acceptable for feeding cattle (ME >MJ/kg DM) (NRC, 2001). Besides, it increases the net energy‐lactation (NE_L_) by 12%. In vivo study confirmed that EFE improves milk production (Lunsin et al., 2021; Mohamed et al., 2013). Also, at this dose, the EFE1 increased in microbial production of crude protein by 7%. This effect may be due to a better use of nutrients by rumen microbes (Getachew et al., 2004), and harmonization between ME and CP in ruminal fermentation (Kaur et al., 2009). This effect is similar to the result of Salem et al. (2015) who noted an increase of microbial crude protein production of corn silage and concentrate supplemented with EFE. VFA are the principal source of energy in ruminants and they reflect the metabolic activity in the rumen (Lee et al., 2018). In this study, EFE1 at medium dose increases the production of volatile fatty acids by 21%. A similar effect was demonstrated in vitro (Sujani et al., 2015) and in vivo (Arriola et al., 2011). The high dose of EFE1 decreases NDF and NDF contents, increases NFC and SDM of BSG. However, it decreases the potential of gas production. The inhibitory effect of EFE on high dose has also been proven in vivo (Lunsin et al., 2021) and in vitro (Abid et al., 2019 a; b). This phenomenon might be explained by the fact that excessive doses of enzymes mask the surface of feed particles, which reduce the microorganisms' adhesion to the substrate and subsequent fermentation (McAllister et al. (2000)). Nsereko et al. (2000) assumed that the extreme doses of this additive can liberate sugar, which remains linked to the fiber, which will possibly trap the places of the action of the enzyme.

## 5 CONCLUSION

These results showed clearly that the mode of action of EFE varies depending on the type of EFE, dose of EFE, and their interactions, which highlights the importance of determining the dose compatibility of each EFE product. In fact, only the EFE_1_ at 2 µl/g dry matter hydrolyzed cell‐wall components, increased solubilized dry matter, stimulated the in vitro fermentation, and increased the nutritional value of the BSG. At 4 µl/ g dry matter, it hydrolyzes cell‐wall components but decreases nutritional value of the BSG.

## CONFLICT OF INTEREST

The authors declare that they have no conflict of interest.

## AUTHOR CONTRIBUTIONS

**Khalil Abid:** Conceptualization (equal); Data curation (equal); Formal analysis (equal); Validation (equal); Visualization (equal); Writing – original draft (equal). **Jihene Jabri:** Data curation (equal); Formal analysis (equal); Supervision (equal). **Hela Yaich:** Supervision (equal). **Atef Malek:** Project administration (equal); Validation (equal). **Jamel Rekhis:** Project administration (equal); Validation (equal). **Mohamed Kamoun:** Methodology (equal); Project administration (equal); Writing – review & editing (equal).

## References

[fsn32743-bib-0001] Abid, K. , Jabri, J. , Ammar, H. , Ben Said, S. , Yaich, H. , Malek, A. , Rekhis, J. , López, S. , & Kamoun, M. (2020). Effect of treating olive cake with fibrolytic enzymes on feed intake, digestibility and performance in growing lambs. Animal Feed Science and Technology, 261, 114405. 10.1016/j.anifeedsci.2020.114405

[fsn32743-bib-0002] Abid, K. , Jabri, J. , Beckers, Y. , Yaich, H. , Malek, A. , Rekhis, J. , & Kamoun, M. (2019). Influence of adding fibrolytic enzymes on the ruminal fermentation of date palm by‐products. Archives Animal Breeding, 62(1), 1–8. 10.5194/aab-62-1-2019 31807609 PMC6852871

[fsn32743-bib-0003] Abid, K. , Jabri, J. , Beckers, Y. , Yaich, H. , Malek, A. , Rekhis, J. , & Kamoun, M. (2019). Effects of exogenous fibrolytic enzymes on the ruminal fermentation of agro‐industrial by‐products. South African Journal of Animal Science, 49(4), 612–618. 10.4314/sajas.v49i4.2

[fsn32743-bib-0004] Arriola, K. G. , Kim, S. C. , Staples, C. R. , & Adesogan, A. T. (2011). Effect of fibrolytic enzyme application to low‐ and high‐concentrate diets on the performance of lactating dairy cattle. Journal of Dairy Science, 94(2), 832–841. 10.3168/jds.2010-3424 21257052

[fsn32743-bib-0005] Association of Official chemists Analytical Chemists (1995). Official methods of analysis of AOAC international (pp. 16, 1–338) Washington DC, USA: AOAC International. http://lib3.dss.go.th/fulltext/scan_ebook/aoac_1995_v78_n3.pdf

[fsn32743-bib-0006] Bailey, M. J. , Biely, P. , & Poutanen, K. (1992). Interlaboratory testing of methods for assay of xylanase activity. Journal of Biotechnology, 23(3), 257–270. 10.1016/0168-1656(92)90074-J

[fsn32743-bib-0007] Beauchemin, K. A. , Colombatto, D. , & Morgavi, D. P. (2004). A rationale for the development of feed enzyme products for ruminants. Canadian Journal of Animal Science, 84(1), 23–36. 10.4141/A02-103

[fsn32743-bib-0008] Beauchemin, K. A. , & Holtshausen, L. (2010). Enzymes in farm animal nutrition. In M. R. Bedford , & G. G. Partridge (Eds). Developments in enzyme usage in ruminants (pp. 8, 2, 206–230) Marlborough Wiltshire UK: CABI.

[fsn32743-bib-0009] Blümmel, M. , Makkar, H. P. S. , & Becker, K. (1997). In vitro gas production: A technique revisited. Journal of Animal Physiology and Animal Nutrition, 77(1–5), 24–34. 10.1111/j.1439-0396.1997.tb00734.x

[fsn32743-bib-0010] Clinquart, A. , Micol, D. , Brundseaux, C. , Dufrasne, I. , & Istasse, L. (1995). Utilisation des matières grasses chez les bovins à l'engraissement. INRA Productions Animales, 8(1), 29–42. 10.20870/productions-animales.1995.8.1.4102

[fsn32743-bib-0011] Díaz, A. , Ranilla, M. J. , Giraldo, L. A. , Tejido, M. L. , & Carro, M. D. (2015). Treatment of tropical forages with exogenous fibrolytic enzymes: Effects on chemical composition and in vitro rumen fermentation. Journal of Animal Physiology and Animal Nutrition, 99(2), 345–355. 10.1111/jpn.12175 24605885

[fsn32743-bib-0012] Duncan, D. B. (1955). Plages multiples et tests F multiples. Biométrie, 11, 1–41. 10.2307/3001478

[fsn32743-bib-0013] Elghandour, M. M. , Antolin‐Cera, X. , Salem, A. Z. , Barbabosa‐Pliego, A. , Valladares‐Carranza, B. , & Ugbogu, E. A. (2018). Influence of Escherichia coli inclusion and soybean hulls based diets on ruminal biomethane and carbon dioxide productions in sheep. Journal of Cleaner Production, 192, 766–774. 10.1016/j.jclepro.2018.05.002

[fsn32743-bib-0014] Elwakeel, E. A. , Titgemeyer, E. C. , Johnson, B. J. , Armendariz, C. K. , & Shirley, J. E. (2007). Fibrolytic enzymes to increase the nutritive value of dairy feedstuffs. Journal of Dairy Science, 90(11), 5226–5236. 10.3168/jds.2007-0305 17954763

[fsn32743-bib-0015] France, J. , Dijkstra, J. , Dhanoa, M. S. , Lopez, S. , & Bannink, A. (2000). Estimating the extent of degradation of ruminant feeds from a description of their gas production profiles observed in vitro: Derivation of models and other mathematical considerations. British Journal of Nutrition, 83(2), 143–150. 10.1017/S0007114500000180 10743493

[fsn32743-bib-0016] Getachew, G. , Blümmel, M. , Makkar, H. P. S. , & Becker, K. (1998). In vitro gas measuring techniques for assessment of nutritional quality of feeds: A review. Animal Feed Science and Technology, 72(3–4), 261–281. 10.1016/S0377-8401(97)00189-2

[fsn32743-bib-0017] Getachew, G. , DePeters, E. , & Robinson, P. (2004). In vitro gas production provides effective method for assessing ruminant feeds. California Agriculture, 58(1), 54–58. 10.3733/ca.v058n01p54.2004

[fsn32743-bib-0018] Han, K. H. , Ko, J. H. , & Yang, S. H. (2007). Optimizing lignocellulosic feedstock for improved biofuel productivity and processing. Biofuels, Bioproducts and Biorefining, 1(2), 135–146. 10.1002/bbb.14

[fsn32743-bib-0019] Kaur, R. , Garcia, S. C. , Fulkerson, W. J. , & Barchia, I. (2009). Utilisation of forage rape (Brassica napus) and Persian clover (Trifolium resupinatum) diets by sheep: Effects on whole tract digestibility and rumen parameters. Animal Production Science, 50(1), 59–67. 10.1071/EA08309

[fsn32743-bib-0020] Kholif, A. E. , Elghandour, M. M. Y. , Rodríguez, G. B. , Olafadehan, O. A. , & Salem, A. Z. M. (2017). Anaerobic ensiling of raw agricultural waste with a fibrolytic enzyme cocktail as a cleaner and sustainable biological product. Journal of Cleaner Production, 142(4), 2649–2655. 10.1016/j.jclepro.2016.11.012

[fsn32743-bib-0021] Lazarevich, A. N. , & Lesnov, A. P. (2010). Brewer's spent grain in pig feeding. Svinovodstvo (Moskva), 8, 46–48.

[fsn32743-bib-0022] Lee, S. J. , Shin, N. H. , Jeong, J. S. , Kim, E. T. , Lee, S. K. , Lee, I. D. , & Lee, S. S. (2018). Effects of Gelidium amansii extracts on in vitro ruminal fermentation characteristics, methanogenesis, and microbial populations. Asian‐Australasian Journal of Animal Sciences, 31(1), 71–79. 10.5713/ajas.17.0619 29295611 PMC5756926

[fsn32743-bib-0024] Lunsin, R. , Pilajun, R. , Cherdthong, A. , Wanapat, M. , Duanyai, S. , & Sombatsri, P. (2021). Influence of fibrolytic enzymes in total mixed ration containing urea‐molasses‐treated sugarcane bagasse on the performance of lactating Holstein‐Friesian crossbred cows. Animal Science Journal, 92(1), e13652. 10.1111/asj.13652 34717034

[fsn32743-bib-0025] Lynch, K. M. , Steffen, E. J. , & Arendt, E. K. (2016). Brewers' spent grain: A review with an emphasis on food and health. Journal of the Institute of Brewing, 122(4), 553–568. 10.1002/jib.363

[fsn32743-bib-0026] Makkar, H. P. (2010). In vitro screening of feed resources for efficiency of microbial protein synthesis. In P. E. Vercoe , H. P. S. Makkar , & A. C. Schlink (Eds.). In vitro screening of plant resources for extra‐nutritional attributes in ruminants: Nuclear and related methodologies (pp. 107–144). Heidelberg: Springer.

[fsn32743-bib-0027] McAllister, T. A. , Stanford, K. , Bae, H. D. , Treacher, R. J. , Hristov, A. N. , Baah, J. , Shelford, J. A. , & Cheng, K. J. (2000). Effect of a surfactant and exogenous enzymes on digestibility of feed and on growth performance and carcass traits of lambs. Canadian Journal of Animal Science, 80(1), 35–44. 10.4141/A99-053

[fsn32743-bib-0028] McDonald, P. , Edwards, R. A. , Greenhalgh, J. F. D. , Morgan, C. A. , Sinclair, L. A. , & Wilkinson, R. G. (2011). Animal nutrition (pp. 7, 1–692) New York: Prentice Hall/Pearson.

[fsn32743-bib-0029] Menke, K. H. , & Steingass, H. (1988). Estimation of the energetic feed value obtained from chemical analysis and in vitro gas production using rumen fluid. Animal Research and Development, 28, 7–55. https://scholar.google.com/scholar_lookup?journal=Anim+Res+Dev&title=Estimation+of+the+energetic+feed+value+obtained+from+chemical+analysis+and+in+vitro+gas+production+using+rumen+fluid&author=KH+Menke&author=H+Steingass&volume=28&publication_year=1988&pages=7‐55&

[fsn32743-bib-0030] Misra, A. K. , Mishra, A. S. , Tripathi, M. K. , Prasad, R. , Vaithiyanathan, S. , & Jakhmola, R. C. (2006). Optimization of solid state fermentation of mustard (Brassica campestris) straw for production of animal feed by white rot fungi (Ganoderma lucidum). Asian‐Australasian Journal of Animal Sciences, 20(2), 208–213. 10.5713/ajas.2007.208

[fsn32743-bib-0031] Mohamed, D.‐D. , Borhami, B. E. , El‐Shazly, K. A. , & Sallam, S. M. A. (2013). Effect of dietary supplementation with fibrolytic enzymes on the productive performance of early lactating dairy cows. Journal of Agricultural Science, 5(6), 146–155. 10.5539/jas.v5n6p146

[fsn32743-bib-0032] Morais, G. , Daniel, J. L. P. , Kleinshmitt, C. , Carvalho, P. A. , Fernandes, J. , & Nussio, L. G. (2017). Additives for grain silages: A review. Slovak Journal of Animal Science, 50(1), 42–54. https://sjas.ojs.sk/sjas/article/view/137

[fsn32743-bib-0033] Mussatto, S. I. (2014). Brewer's spent grain: A valuable feedstock for industrial applications. Journal of the Science of Food and Agriculture, 94(7), 1264–1275. 10.1002/jsfa.6486 24254316

[fsn32743-bib-0034] Mussatto, S. I. , Dragone, G. , & Roberto, I. C. (2006). Brewers' spent grain: Generation, characteristics and potential applications. Journal of Cereal Science, 43(1), 1–14. 10.1016/j.jcs.2005.06.001

[fsn32743-bib-0035] National Research Council (2001). Nutrient requirements of dairy cattle. 7(1–405). Washington, USA: National Academies Press. 10.17226/9825 38386771

[fsn32743-bib-0036] Nsereko, V. L. , Morgavi, D. P. , Rode, L. M. , Beauchemin, K. A. , & McAllister, T. A. (2000). Effects of fungal enzyme preparations on hydrolysis and subsequent degradation of alfalfa hay fiber by mixed rumen microorganisms in vitro. Animal Feed Science and Technology, 88(3–4), 153–170. 10.1016/S0377-8401(00)00225-X

[fsn32743-bib-0037] Salem, A. Z. M. , Buendía‐Rodríguez, G. , Elghandour, M. M. M. , Berasain, M. A. M. , Jiménez, F. J. P. , Pliego, A. B. , Chagoyán, J. C. V. , Cerrillo, M. A. , & Rodríguez, M. A. (2015). Effects of cellulase and xylanase enzymes mixed with increasing doses of Salix babylonica extract on in vitro rumen gas production kinetics of a mixture of corn silage with concentrate. Journal of Integrative Agriculture, 14(1), 131–139. 10.1016/S2095-3119(13)60732-7

[fsn32743-bib-0038] SAS Institute Inc . (2011). SAS/STAT^®^ 9.3. User’s Guide. (1–8640). SAS Institute Inc. http://support.sas.com/documentation/onlinedoc/stat/930/

[fsn32743-bib-0039] Sujani, S. , Pathirana, I. , & Seresinhe, T. (2015). Enhanced in vitro fermentation parameters of guinea grass ecotype “A” (Panicum maximum) and rice straw (Oryza sativa) with supplementation of exogenous fibrolytic enzymes. Livestock Research for Rural Development, 27(3), 41. http://www.lrrd.org/lrrd27/3/suja27041.html

[fsn32743-bib-0040] Van Soest, P. V. , Robertson, J. B. , & Lewis, B. (1991). Methods for dietary fiber, neutral detergent fiber, and nonstarch polysaccharides in relation to animal nutrition. Journal of Dairy Science, 74(10), 3583–3597. 10.3168/jds.S0022-0302(91)78551-2 1660498

[fsn32743-bib-0041] Varga, G. A. , & Kolver, E. S. (1997). Microbial and animal limitations to fiber digestion and utilization. The Journal of Nutrition, 127(5), 819S–823S. 10.1093/jn/127.5.819S 9164244

[fsn32743-bib-0042] Wanapat, M. , Ampapon, T. , Phesatcha, K. , & Kang, S. (2018). Effect of banana flower powder on rumen fermentation, synthesis of microbial protein and nutrient digestibility in swamp buffaloes. Animal Production Science, 59(9), 1674–1681. 10.1071/AN18063

[fsn32743-bib-0043] Wang, Y. , McAllister, T. A. , Rode, L. M. , Beauchemin, K. A. , Morgavi, D. P. , Nsereko, V. L. , Iwaasa, A. D. , & Yang, W. (2001). Effects of an exogenous enzyme preparation on microbial protein synthesis, enzyme activity and attachment to feed in the Rumen Simulation Technique (Rusitec). British Journal of Nutrition, 85(3), 325–332. 10.1079/bjn2000277 11299078

[fsn32743-bib-0044] Wood, T. M. , & Bhat, K. M. (1988). Methods for measuring cellulase activities. Methods in Enzymology, 160, 87–112. 10.1016/0076-6879(88)60109-1

[fsn32743-bib-0045] Yang, W. Z. , Beauchemin, K. A. , & Rode, L. M. (1999). Effects of an enzyme feed additive on extent of digestion and milk production of lactating dairy cows. Journal of Dairy Science, 82(2), 391–403. 10.3168/jds.S0022-0302(99)75245-8 10068960

